# Double counting of clients using services in Iran: implications for assessing the reach of harm reduction programs

**DOI:** 10.1186/s12954-023-00851-5

**Published:** 2023-08-16

**Authors:** Fatemeh Tavakoli, Willi Mcfarland, Nima Ghalekhani, Mehrdad Khezri, Ali Akbar Haghdoost, Mohammad Mehdi Gouya, Marzieh Mahboobi, Ali Mohammad Hosseionpour, Ali Komasi, Mehdi Ghorbanian, Nasim Nasiri Moghadam, Maryam Taghipour, Hamid Sharifi

**Affiliations:** 1 HIV/STI Surveillance Research Center, and WHO Collaborating Center for HIV Surveillance, Institute for Futures Studies in Health, Kerman University of Medical Sciences, Kerman, Iran; 2 Department of Epidemiology and Biostatistics, University of California San Francisco, San Francisco, CA USA; 3 Department of Epidemiology, School of Global Public Health, New York University, New York, NY USA; 4 Department of Infectious Disease and Tropical Medicine, School of Medicine, Iran University of Medical Sciences, Tehran, Iran; 5 Iranian Center for Communicable Diseases Control, Ministry of Health and Medical Education, Tehran, Iran; 6 Mashhad University of Medical Sciences, Mashhad, Iran; 7 Kermanshah University of Medical Sciences, Kermanshah, Iran; 8 Alborz University of Medical Sciences, Alborz, Iran; 9 Center for HIV/STI Control and Prevention, Kerman University of Medical Sciences, Kerman, Iran; 10 Shiraz University of Medical Sciences, Shiraz, Iran

**Keywords:** Harm reduction, Double counting, HIV, Drug use, Iran

## Abstract

**Background:**

Many people with high-risk sexual or injection behaviors use harm reduction services with different identities and are therefore counted more than once in client databases. This practice results in inaccurate statistics on the number of clients served and the effective reach of these services. This study aimed to determine the levels of double counting of clients of harm reduction services, including needle and syringe programs, condom distribution, HIV testing and counseling, and methadone maintenance in five cities in Iran.

**Methods:**

Between September and March 2020, our study included 1630 clients, 115 staff of harm reduction centers, and 30 experts in the field of harm reduction in five cities in Iran. Clients of harm reduction services were asked about using harm reduction services multiple times at the same center or at different centers in the last year using different identities. Estimates of double counting derived from client responses were validated by panels of center staff and experts in harm reduction.

**Results:**

Synthesizing data from clients, staff, and experts, the final estimates of double counting of clients using harm reduction services were: HIV testing 10% (95% confidence interval [CI] 0–15), needle and syringe programs 17% (95% CI 8.5–20), condom distribution programs 13% (95% CI 3–19), HIV/STI counseling 10% (95% CI 0–16), and methadone maintenance 7% (95% CI 2–10).

**Conclusion:**

Double counting of clients in harm reduction services in Iran is substantial. Data on clients reach by harm reduction services need to be corrected for double counting to improve program planning, client population size estimation, and efficient resource allocation.

## Background

Harm reduction programs provide services to help decrease the risk of diseases when eliminating causal behaviors, such as sharing injection equipment, is not yet possible for some clients [1, 2]. For example, the World Health Organization (WHO), the Joint United Nations Programme on HIV/AIDS (UNAIDS), and the United Nations Office on Drugs and Crime (UNODC) recommend harm reduction programs as best practices for the prevention of HIV among people who inject drugs (PWID) [3]. These best practice harm reduction programs include needle and syringe programs, opioid agonist therapy (OAT), HIV testing and counseling (HTC), condom promotion programs, targeted information, and education and communication [3]. These interventions have evidence supporting their efficacy in preventing the spread of HIV as well as mitigating other harm associated with drug use [3, 4].

Iran has a high prevalence of drug use in the adult population, with many individuals having a history of injecting drugs [5]. While smoking opium has traditionally been used in Iran, there has been a shift in drug use patterns in recent decades, including an increased number of people who inject drugs (PWID) [6, 7]. Based on available evidence, it is estimated that there are approximately 345,000 PWID (95% confidence interval [CI] 329,000–363,000) in Iran of whom 243,000 (95% CI 227,000–257,000) are frequent users [5]. A systematic review conducted among female sex workers (FSWs) in Iran found that the prevalence of both injection (10.7%), and non-injection drug use (76.1%) among FSWs is high [6]. These findings highlight the very high numbers of drug users in Iran and underscore the need to reach large numbers of clients with effective harm reduction and substance use treatment services [11].

Iran made harm reduction programs available as an official policy in 2005. Iran developed the most robust harm reduction infrastructure in the Middle East, marked by the availability of low threshold methadone maintenance treatment (MMT), clean needle and syringe programs, condom distribution services, and training sessions on how to prevent HIV and other blood-transmitted diseases [7, 8]. Harm reduction services in Iran are mainly provided by mobile teams, drop-in centers (DICs), voluntary counseling and testing centers (VCTs), night shelters, and addiction treatment centers [9]. Substance use treatment services are often offered within harm reduction programs. The effectiveness of existing policies and program can be assessed in how successful they are in providing individual health benefits and public benefits such as better quality of life, crime reduction, and safety [10].

Nevertheless, harm reduction programs in Iran face many challenges. One challenge is monitoring and evaluating the reach, coverage, and intensity of services provided to clients [11]. Due to a lack of an integrated information system between programs, individuals can receive services from multiple centers or from one service using different identities. Moreover, due to confidentiality concerns, true identities may not be provided by clients or recorded by programs. These challenges result in double counting of the number of clients served by programs and therefore inaccurate statistics on the reach of programs for the target population [12]. Policymakers need client-based counts rather than service-based counts to evaluate program coverage, effectively allocate resources, and reduce waste do to duplicate services. Unfortunately, the extent of double counting of clients is seldom quantified. Therefore, this study aims to determine the double counting of clients using harm reduction services in several cities of Iran to guide correction factors needed to achieve more accurate information for the health system.

## Methods

Following approvals from health system authorities at the provincial level, the study was implemented between September and March 2020. The study participants were drawn from clients and staff of harm reduction centers, including mobile centers, DICs, and addiction treatment centers located in five cities in different regions of Iran. These cities were Kerman, Kermanshah, Mashhad, Shiraz, and Karaj. This approach was taken to ensure the inclusion of diverse geographic regions in the study. We used panels of experts to corroborate findings from the client and staff interviews. We evaluated double counting at several different sexual and injection harm reduction services, including HIV rapid testing, needle and syringe programs, programs providing free condoms, HIV/STI counseling, and methadone maintenance using a brief questionnaire or checklist. The clients and staff of harm reduction centers within the defined study period were asked to complete the checklist after providing informed consent. Outreach teams were used to access clients and staff of mobile programs present in hotspots in each city.

We administered separate checklists to clients and staff. The client checklist included questions such as “Have you received harm reduction services, such as HIV testing, from Center A in the last 12 months?” and "Have you received these services from Center B in the last 12 months?" If clients responded affirmatively, they were asked to indicate the number of times they received services and whether the identity they gave the service provider was different or the same on each occasion. In parallel, staff checklists asked: "What is your estimate of the proportion of service recipients who provide different identities when receiving services from different harm reduction centers?" and "In your opinion, what proportion of service recipients do you think are receiving the necessary services from different centers?".

We defined the double counting in the use of services at two levels: (1) double counting of clients within each service center at different times in the last year; and (2) double counting of clients across different service centers in the last year. The estimates of these two sources of double counting as reported by clients and staff were validated against an expert panel in each city. For the estimate of double counting of the same client within the same center at different times, the numerator was the number of people who have received services more than once using a different identity and the denominator was all people who have received services at the same center. For the estimate of double counting of the same client at different centers, the numerator was the number of people who received one specific service at more than one center and the denominator was all people who have received the specific service.

We combined the results of clients and staff sources. In this approach, if the clients' opinion had a larger estimation, that number was considered as the best estimation. Otherwise, the average of the two estimates was considered as the combined estimate of clients and staff. The median of the numbers related to different cities based on each service was used to estimate the percentage of people with different identities at each center and the percentage of people with different identities at other centers.

**Fig. 1:**
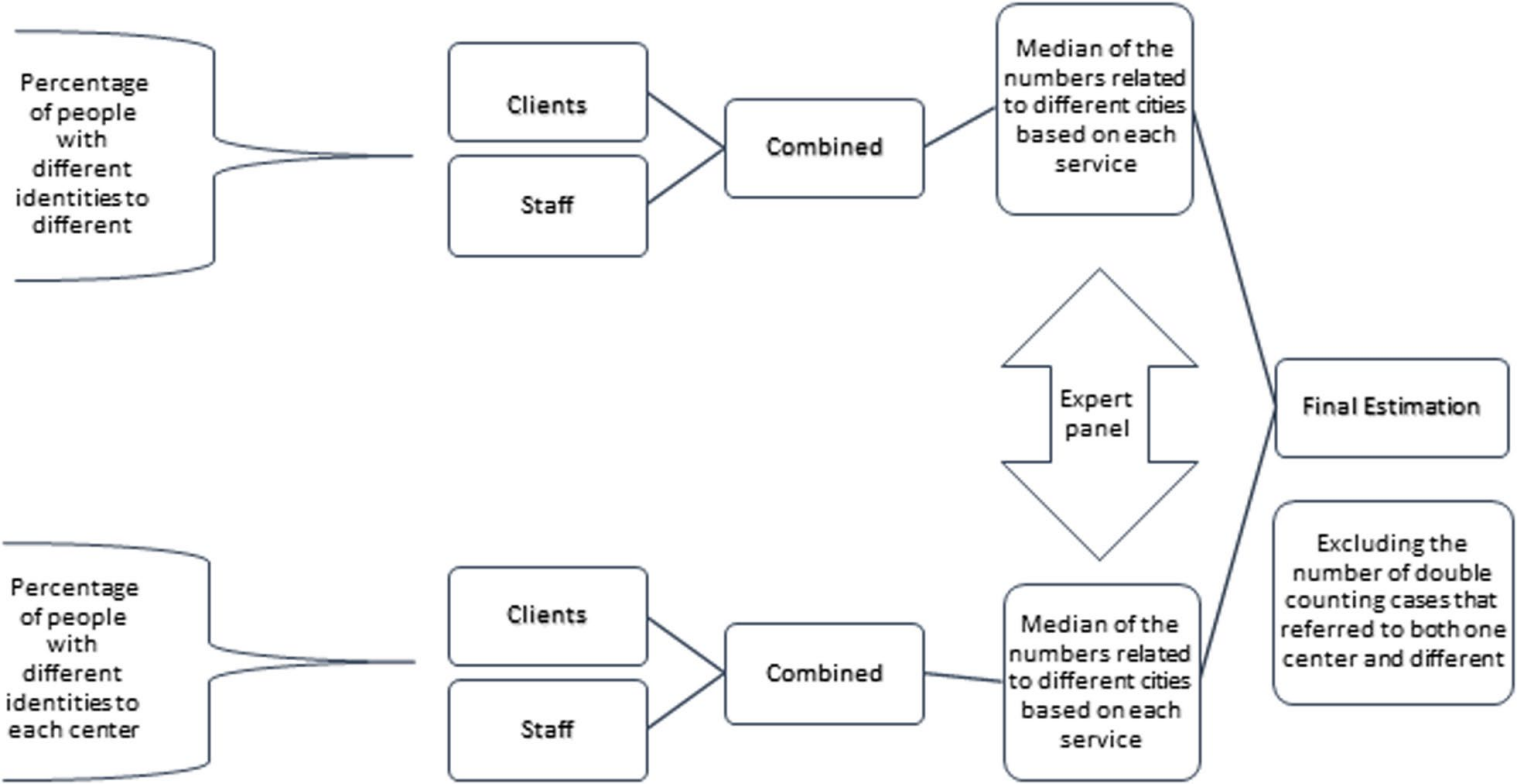
Flow diagram of final estimates of the percentage of people with different identities to one or more harm reduction centers in Iran, 2020

Following the above estimation, a panel was convened comprising experts in harm reduction service delivery as key informants in each city. At the panel meeting, we asked each member to give their best estimate for the percentage of people presenting for services using different identities within each center, and the percentage of people using different identities presenting to different centers. The panel discussed the rationale for their estimates. The median of the numbers related to different cities based on each service was used for the final estimations (Fig. 1).

To calculate the final combined estimates, the percentage of people with different identities to any center or other centers was considered. The number of double-counting cases that referred to both one center and different centers was excluded. We also used the opinion of an expert panel to cross-validate the final combined estimates. To calculate the 95% CI, we considered the lowest estimate of each service as the lower limit and the highest estimate as the upper limit of CI.

## Results

### Participants

A total of 1630 clients were interviewed, including 286 in Kerman, 247 in Kermanshah, 435 in Mashhad, 442 in Shiraz, and 220 in Karaj. The mean age of clients was 43.0 years (SD 12.3) and 69.7% were male (Table 1). A total of 115 staff were recruited from the same cities, including 17 in Kerman, 17 in Kermanshah, 35 in Mashhad, 34 in Shiraz, and 12 in Karaj. Five to six experts participated in the panels for each city.

**Table 1:** Socio-demographic characteristics of study participants, clients of harm reduction service centers, Iran, 2020

Variables	
Overall	1630
	Mean [SD]
Age in years	43.0 (12.3)
Age at first drug use, years	22.3 (6.8)
Age at first injection, years	30.0 (8.2)
Age at first sexual intercourse, years	18.8 (9.7)
	n (%)
*Sex*	
Male	1111 (69.7)
Female	483 (30.3)
*Marital status*	
Single	360 (23.0)
Married	473 (30.2)
Divorced/widowed	733 (46.8)
*Mode of first drug use*	
Injection	120 (8.4)
Non-injection	1306 (91.6)
*Needle or syringe sharing at last injection*	
Yes	85 (10.9)
No	697 (89.1)
*Condomless sex with any sexual partner at last sex act*	
Yes	717 (63.2)
No	417 (36.8)
*Received sterile needle/syringe in the last 12 month*	
Yes	1161 (72.84)
No	433 (27.16)

### Double counting within each service center

Based on the opinion of clients and staff, the percentage of double counting of using services within each service center at different times was estimated for HIV testing at 3% (95% CI 1–10), needle and syringe programs at 9% (95% CI 5.5–12), condom distribution at 6% (95% CI 2–10), HIV/STI counseling at 2.5% (95% CI 1–10), and methadone maintenance at 1.5% (95% CI 0–25). According to the expert panels, double counting in HIV testing services was estimated at 4% (95% CI 1–10), needle and syringe programs at 6% (95% CI 2–10), condom distribution programs at 4% (95% CI 1–7), HIV/STI counseling at 1% (95% CI 0–2), and methadone maintenance at 0% (95% CI 0–3).

### Double counting across different service centers

Based on the opinion of clients and staff, double counting of using services at different service centers was estimated for HIV testing at 8% (95% CI 0–10), needle and syringe programs at 10% (95% CI 3.5–10), condom distribution programs at 10% (95% CI 2–15), HIV/STI counseling at 8% (95% CI 0–10), and methadone maintenance treatment at 5% (95% CI 1–21) (Table 2). Based on the expert panels, double counting at different HIV testing programs was estimated at 9% (95% CI 1–20), needle and syringe programs at 10% (95% CI 2–30), condom distribution programs at 10% (95% CI 2–20), HIV/STI counseling at 7% (95% CI 1–10), and methadone maintenance treatment at 3% (95% CI 0–40).

**Table 2:** Percentage of double counting of using services in each service center at different times and different service centers based on responses of clients, staff, and an expert panel, Iran, 2020

Services	Percentage of double counting of using services in each service center at different times		Percentage of double counting of using services in different service centers		Overall double counting estimation of using services in the same center at different times or different centers
	Clients and staff % (CI^a^)	Expert panel % (CI)	Clients and staff (CI)	Expert panel (CI)	Clients and staff % (CI)
HIV testing	3 (1–10)	4 (1–10)	8 (0–10)	9 (1–20)	10 (0–15)
Needle and syringe programs	9 (5.5–12)	6 (2–10)	10 (3.5–10)	10 (2–30)	17 (8.5–20)
Condom distribution programs	6 (2–10)	4 (1–7)	10 (2–15)	10 (2–20)	13 (3–19)
HIV/STI counseling	2.5 (1–10)	1 (0–2)	8 (0–10)	7 (1–10)	10 (0–16)
Methadone maintenance	1.5 (0–25)	0 (0–3)	5 (1–21)	3 (0–40)	7 (2–10)

^a^95% confidence intervals

### Synthesized estimates

**Fig. 2:**
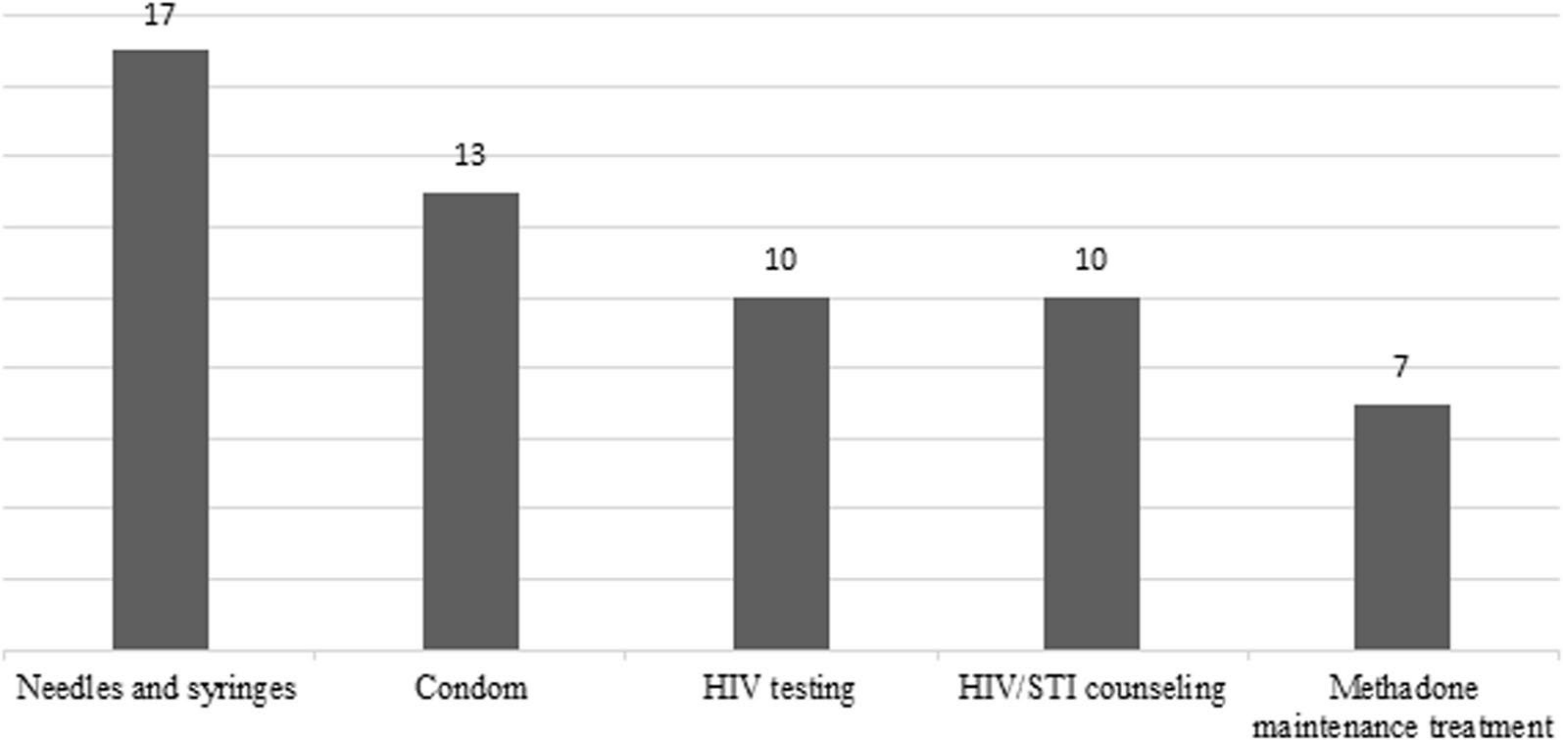
Final estimates of the percentage of people with different identities to one or more harm reduction centers in Iran, 2020

The final synthesized estimation of double counting of using harm reduction services were estimated for HIV testing at 10% (95% CI 0–15), needle and syringe programs at 17% (95% CI 8.5–20), condom distribution at 13% (95% CI 3–19), HIV/STI counseling at 10% (95% CI 0–16), and methadone maintenance treatment at 7% (95% CI 2–10) (Fig. 2).

## Discussion

This study estimates that the double counting of clients using harm reduction services in Iran may be substantial. Needle and syringe programs had the highest percentage of double counting of using services within each harm reduction center at different times and for using services in different harm reduction centers. Methadone maintenance services had the lowest percentage of double counting. Our findings are robust in that they are based on the reports of a large sample of clients, the program staff, and experts in the field. To the best of our knowledge, this is the first study to estimate double counting of clients in harm reduction services in Iran. We consider several possible reasons for the double counting. First, the high mobility of the target population of drug users may result in double counting, particularly between different centers. Second, some services can be obtained anonymously or without verification of identity. Third, the services of one center may become unavailable at different times or may be limited in number per client, and therefore incentivizing people to provide different identities at the same site at different times or at different centers.

Our findings of substantial double counting of clients affect the assessment of meeting prevention targets for the reach and coverage of harm reduction programs. For example, the WHO recommends providing 300 clean needles and syringes per PWID per year by 2030 to effectively tackle parenteral HIV transmission [13]. By 2016, only 12 countries provided at least 200 clean needles per PWID per year, and Iran only provided 50 syringes and needles per PWID per year [14]. While the number of needles and syringes provided to PWID in Iran is already low compared to the target, our results show that there may be a 17% double counting. Thus, the actual number of PWID served will be even lower than reported. Service providers should consider double counting when setting targets for clients reached and reporting progress towards these targets. The effectiveness of harm reduction programs depends on their reach and intensity. For example, supplying clean needles and syringes has been demonstrated to reduce heroin use, associated deaths, HIV risk behaviors, and criminal activity [15]. In a study by Hurley et al. [16], HIV prevalence increased by 5.9% per year in cities without needle and syringe programs and decreased by 5.8% per year in cities with such programs. Evaluations by the WHO found that needle and syringe exchange is effective in preventing the spread of HIV, lacks negative consequences, is cost-effective relative to other interventions (even resulting in cost-savings), and has positive externalities such as reduced crime [13, 17, 18]. With respect to preventing sexual transmission of HIV, data for condom distribution for PWID is scant for the region but indicate low coverage overall [19]. Our study suggests an over-estimation given the 13% double counting in providing condoms to people through harm reduction centers.

We found that methadone services had the lowest level of double counting within one or across multiple service centers. The requirement of presenting national identity cards to receive methadone at harm reduction centers is likely to prevent PWID from presenting with different alias at the same or different centers. Moreover, there is a centralized Iranian Drug Abuse Treatment Information System (IDATIS) that verifies the recipients of methadone maintenance services for drug users throughout the country, also based on valid national identification numbers [20].

The use of unique codes can reduce double counting of clients at other harm reduction services in Iran. For example in Uzbekistan, Tajikistan, and the Ferghana Valley region of Kyrgyzstan, a standardized method provides each client with a unique identifier code (UIC) that can be applied across multiple service centers. Services in different countries have developed various but similar UIC systems [21, 22]. The UIC is composed of non-identifying elements that are created in the same manner each time, with easy recall by clients, without revealing their identity, and without the ability to decode. A UIC can reveal whether a client is reached regularly by a service, such as a needle and syringe program, without collecting personally identifying information such as names or government-issued national identification numbers. The UIC enables data to de-duplicate client counts and track services used by individual clients’ within and across programs.

The findings presented here should be interpreted with caution due to limitations. First, we do not have accurate population size estimations for PWID or other at-risk groups using harm reduction services. Second, although efforts were made to recruit individuals from diverse geographical areas, this sample is not representative of all of Iran. Nonetheless, we chose cities with regional and geographical diversity to improve generalization. Third, recall bias may affect results as interviewing required participants to reflect on multiple past events. Fourth, it was not possible to calculate the potential double counting of participants who may have received services at different types of harm reduction centers or delivery modes, which may have varied between centers.

## Conclusions

We estimate that there is a significant amount of double counting and therefore overestimation in the coverage of harm reduction services in Iran. Policymakers should consider this double counting when setting targets and reporting services delivered. Our findings provide an approach to establishing correction factors for reporting harm reduction services utilization in diverse settings worldwide.

## Data Availability

Data will be available upon request submitted to the corresponding author (sharifihami@gmail.com).
